# Repetitive transcranial magnetic stimulation combined with respiratory muscle training for pulmonary rehabilitation after ischemic stroke—A randomized, case-control study

**DOI:** 10.3389/fnagi.2022.1006696

**Published:** 2022-09-23

**Authors:** Haiyan Cao, Xiaoming Chen, Xuyan Ren, Zhiguo Chen, Chuandao Liu, Jianqiang Ni, Haoyu Liu, Yingjie Fan, Dandan Xu, Huaping Jin, Jie Bao, Huang Yulun, Min Su

**Affiliations:** ^1^Department of Physical Medicine and Rehabilitation, Dushu Lake Hospital of Soochow University, Suzhou, China; ^2^Kunshan Rehabilitation Hospital, Suzhou, China; ^3^Institute of Rehabilitation, Soochow University, Suzhou, China; ^4^The First Affiliated Hospital of Soochow University, Suzhou, Jiangsu, China; ^5^School of Physical Education and Sports Science, Soochow University, Suzhou, China

**Keywords:** respiratory muscle weakness, pulmonary dysfunction, transcranial magnetic stimulation, respiratory muscle training, ischemic stroke

## Abstract

Respiratory muscle weakness often occurs after stroke, which can lead to pulmonary dysfunction (PD). Pulmonary dysfunction prolongs the length of hospital stay and increases the risk of death. In a prospective, randomized, case-control study, we used musculoskeletal ultrasonography (MSUS), and pulmonary function tester to objectively evaluate the efficacy of repetitive transcranial magnetic stimulation (rTMS) combined with respiratory muscle training (RMT) in the treatment of PD in patients with acute ischemic stroke. Sixty-two stroke patients with PD were recruited and eventually 60 patients participated in this study. The control group was treated with RMT, and the treatment group was treated with rTMS on the basis of RMT. Treatment occurred five times a week for 8 weeks. Before and after treatment, diaphragmatic thickness (DT), diaphragmatic thickening fraction (DTF) and diaphragmatic mobility (DM) in patients, bilateral chest wall were measured by MSUS. Meanwhile, FVC, FEV1, FEV1/FVC, PEF, and MVV tested by pulmonary function tester was used to evaluate the improvement of lung functional. activities of daily living (ADL) was used as an objective criterion to evaluate the overall functional recovery of patients before and after treatment. After treatment, DT, DTF, and DM values improved significantly in both the affected and unaffected sides. The FVC, FEV1, FEV1/FVC, PEF, MVV, and ADL were all increased after the treatment. Combined treatment showed a stronger increase than that by RMT treatment alone. The study preliminarily shows that rTMS and RMT could improve lung functional after acute ischemic stroke.

## Introduction

Stroke can cause severe dysfunction (Hreha et al., 2020). Up to 50% of stroke patients experiencing long-term disability, and up to 30% of people are still unable to take care of themselves 6 months after a stroke (Writing Group Members et al., 2012). The research on functional recovery after stroke mainly focused on motor recovery of the hemiplegic limb, language, cognitive and dysphagia recovery. Too little attention was paid to the lung function recovery (Miryutova et al., 2019; Li et al., 2022). Studies have proved that the respiratory muscle strength of the affected side also decreases after stroke (Ramos et al., 2020). The weakness of respiratory muscle shows low endurance during exercise and independent walking ability (Morisawa et al., 2021). Respiratory muscle weakness in stroke patients is usually manifested as pulmonary infection, respiratory failure, atelectasis or sleep disorders (Jung et al., 2014; Liaw et al., 2020; Bruce et al., 2022). If not intervened early, it will progress to restrictive respiratory disease (Lima et al., 2014). They may be due to the intervention of the respiratory control center or respiratory mechanical changes caused by respiratory muscle weakness (Rochester and Mohsenin, 2002 Diaphragmatic dysfunction after stroke is another important factor leading to lung dysfunction, with an incidence of 51.7% (Fabero-Garrido et al., 2022). About 70% of these patients will develop severe respiratory disease (Catalá-Ripoll et al., 2020). It has been reported that the healthy side diaphragm deviation in stroke patients is greater than that of the affected side (Liaw et al., 2020; Yoon et al., 2020). Meanwhile, Lanini et al. (2003) studied the spontaneous breathing of 8 stroke patients. The results showed that the affected hemithorax respiratory movement reduced during voluntary hyperventilation.

Respiratory muscle training (RMT) can improve the lung function of stroke survivors (Yoo and Pyun, 2018). A review study suggests that inspiratory muscle training is more helpful to improve the quality of life and cardiopulmonary fitness of stroke patients (Xiao et al., 2012). In addition, studies have shown that lower respiratory intensity increases the risk of stroke (Yoon et al., 2020).

As a non-invasive *in vitro* neuromodulation technique, rTMS has received much attention from researchers since its birth in 1985 (Matsuda et al., 2021). Research shows that magnetic stimulation can be applied to human respiratory motor cortex (Nierat et al., 2015; Cember et al., 2022). The cortex of rTMS induced unilateral diaphragmatic response is located in the contralateral brain region, 3 cm outside the midline and 2–3 cm before the ear plane (Maskill et al., 1991). Studies have been shown to increase regional blood flow in the bilateral primary motor cortex as defined by rTMS during volitional breathing (Colebatch et al., 1991). So we hypothesize that rTMS may improve the lung function and promote the activities of daily living (ADL) in hemiplegic patients.

We aimed at exploring the effects of rTMS combined with RMT on the lung function in patients with cardiopulmonary dysfunction after acute ischemic stroke. The lesions in the stroke patients we selected were located in the anterior circulation of the brain. Compared with single RMT, rTMS combined with RMT may obtain better therapeutic effect from central and peripheral pathways.

## Materials and methods

### Participants

Patients hospitalized in the rehabilitation department of the Dushu Lake Hospital of Soochow University from June 2019 to June 2020 were screened. The inclusion criteria were described as follows: (1) The first incidence of ischemic stroke with the lesion at the anterior cerebral circulation confirmed by the head MRI scan. (2) Presence of pulmonary dysfunction (less than 80% of the predicted value) assessed by spirometry. (3) Without aphasia or cognitive dysfunction. (4) Vital signs stable. Exclusion criteria: (1) History of lung, chest and abdominal disease. (2) History of smoking. (3) Combined with myasthenia gravis or phrenic nerve palsy. (4) Combined with severe heart disease, liver cirrhosis, renal failure, severe systemic illness and history of malignant disease. (5) Metallic implants in the body.

### Study design

The study is a prospective, randomized, case-control study. The subjects were numbered in the order of inclusion and divided into control and treatment groups by random number tables. Patients voluntarily participated in the experiment and signed informed consent. The study was approved by the ethics committee of the Dushu Lake Hospital of Soochow University. Collect basic information of patients, including age, gender, stroke duration and affected side. Assess the patient’s ADL score. The above work is collected and analyzed by the same person.

### Treatment

Both groups received conventional rehabilitation and treated by one therapist. Conventional rehabilitation training includes respiratory training and limb function training. Each time, for 40 min. Breathing training is to blow balloons and blow bubbles. Limb function training, such as muscle strength training, body position transfer, walking training, activities of daily life training, etc. The control group received threshold RMT (POWERbreathe^®^, International Ltd., Warwickshire, UK) twice a day, 10 min each time in the morning and afternoon, with an interval of 3 h. The initial intensity was 30% of the maximum inspiratory pressure (MIP) and increased by 5% every week until the training intensity reached 60% MIP. Continue to practice with 60% MIP for 2 weeks.

The treatment group used a magnetic stimulator (MagPro R30, Medtronic A/S, Denmark) connected with a 75 mm figure-of-eight water-cooled coil (MCF-B65) for stimulation. The patient wore an International 10–20 system positioning cap. The stimulation site was located 3 cm lateral to the midline and 2–3 cm anterior to the auricular plane. The frequency of 5 Hz with the stimulus intensity was set at 30% above the unaffected side diaphragmatic motor threshold applied (Urban et al., 2002). Patients received 4,200 rTMS pulses per day for 10 min. The RMT was performed after 3 h. Five times a week for 8 weeks. During the treatment the participants were seated in their own wheelchairs. The rTMS, in general, has no special side effects. However, if not used properly, it can cause seizures or syncope, so the treatments were performed by two senior physiotherapists, who observed the patient closely during the treatment process to avoid accidents. One patient in the control group failed to adhere to training due to a new cerebral infarction. With no patient withdrew from the treatment group.

### Clinical evaluations

A portable diagnostic ultrasound system (M-Turbo, ICTx, SonoSite, America) connected with a 6–13 MHz linear array transducer was used to assess DT, DTF and with a 3.0–5.5 MHz transducer was used to assess DM in patients, bilateral chest wall.

#### Diaphragm thickness

**FIGURE 1:**
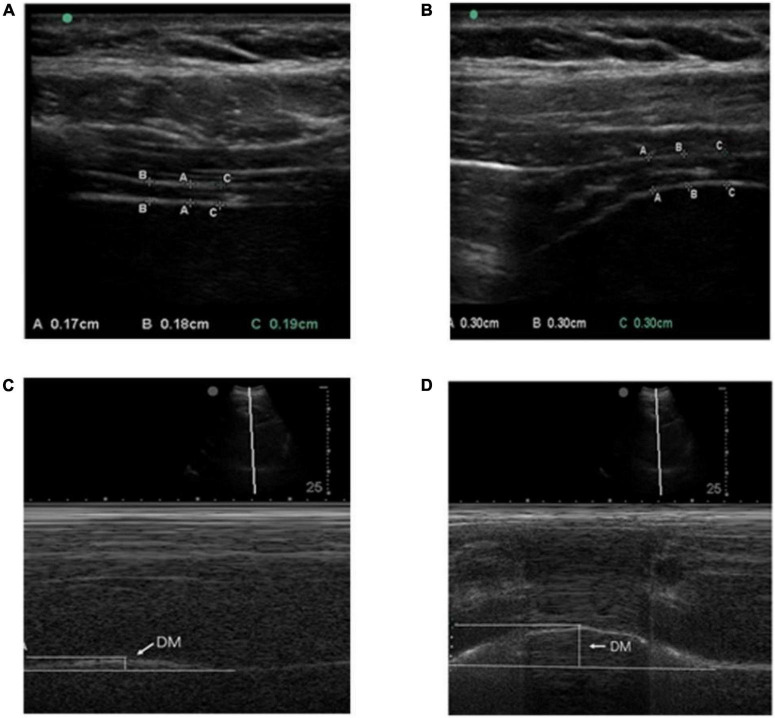
**(A,B)** Respectively indicate the method of measuring the diaphragm thickness of calm end-expiratory and maximum end-inspiratory with the linear array probe. The diaphragm was identified as a three-layered structure, namely the hyperechoic area on both sides (pleural layer and peritoneum) and the middle mixed echo area (composed of anechoic diaphragmatic muscle tissue and hyperechoic fascia). DT is represented by the vertical line between the pleural layer and peritoneum; **(C,D)** respectively, indicates diaphragm mobility during quiet and deep breathing on the M-mode screen. DM is represented by the vertical axis between the line passing through the end of the normal expiration and inspiratory peaks.

The participants in a supine position with spontaneous breathing. Take the linear array probe and place it on the right anterior axillary line. The probe is perpendicular to the 8th–9th intercostal space. If the diaphragm cannot be seen at this position, the probe can be moved up to the 7th–8th intercostal space. The diaphragm is a 3-layer structure under diagnostic sonography. The three-layer structure includes the hyperechoic area of the upper and lower layer (pleural layer and peritoneal layer), and the hypochoechoic middle muscular layer (composed of anechoic diaphragmatic muscle tissue and hyperechoic fascia). Move the cursor to measure the calm end-expiratory diaphragm thickness and the maximum end-inspiratory diaphragm thickness. Values were measured for 3 breathing cycles and averaged (Figures 1A,B). The change in diaphragm thickness from calm end-expiratory to maximum end-inhalation can be calculated, that is, diaphragm thickening rate = (maximum end-inspiratory diaphragm thickness-end-expiratory thickness)/maximum end-expiratory thickness. The measurement method is the same on the left and right sides.

#### Diaphragm mobility

The participants in a supine position with spontaneous breathing. A 2D mode was used to find the best exploration line for each hemidiaphragm. The right used liver as a window, while the spleen was used on the left hemidiaphragm. For right hemidiaphragm, the ultrasound probe was placed at the lower costal margin between the midclavicular and anterior axillary lines. Diaphragm movements were recorded in M-mode and the sampling line should be perpendicular to the diaphragm. Diaphragm movements were recorded during quiet breathing (QB) and deep breathing (DB) (Figures 1C,D). The measurement method is the same on the left and right sides.

Pulmonary function tests by standard spirometry. The Participants take seats. The main indicators of observation including FVC, FEV1, FEV1/FVC, PEF, and MVV using an Master-Screen spirometer (Leibinzstrasse7, 97204 Hoechberg, Germany).

### Statistical analysis

Used Spss20.0 for statistical analysis of all experimental data, values are presented as mean ± standard deviation (X ± SD). For the comparison of data before and after treatment within the group, the paired sample *t*-test was used for those that conformed to the normal distribution, and the non-parametric Wilcoxon rank-sum test was used for the data that did not conform to the normal distribution. The data before and after treatment in the two groups were compared, and the independent samples *t*-test was used for those that conformed to the normal distribution, and the non-parametric Wilcoxon rank-sum test was used for those that did not conform to the normal distribution. *P* < 0.05 representatives have significant differences.

## Results

### General information

Sixty-two patients participated in the experiment, excluding one patient with new cerebral infarction who did not complete the experiment, and 61 patients completed the experiment. Finally, 60 patients aged 26–80, with an average age of 60.7 years, a duration of 4–90 days and an average duration of 30.7 days were eligible for the study. Before treatment, there was no difference in gender (female/male), age, duration, affected side, ADL score, FVC, FEV1, FEV1 \/FVC, PEF, and MVV between the two groups (*P* > 0.05) (see Table 1).

**TABLE 1:** Anthropometric data, ADL, and pulmonary function test results.

	Control group	Treatment group
	
	*n* = 30	*n* = 30
Age (yr)	59.13 ± 11.55	62.27 ± 13.17
Gender (female/male)	1.43 ± 0.50	1.40 ± 0.50
Duration	29.30 ± 24.00	32.10 ± 19.46
Hemiplegia	1.53 ± 0.51	1.40 ± 0.50
ADL	28.50 ± 9.21	27.83 ± 8.17
FVC (L)	2.05 ± 0.57	2.04 ± 0.44
FEV1 (L)	1.16 ± 0.34	1.21 ± 0.27
FEV1/FVC (%)	56.69 ± 3.00	59.52 ± 6.47
PEF (L)	2.69 ± 1.03	2.39 ± 0.70
MVV (L)	53.81 ± 12.60	54.94 ± 12.22

Values are presented as mean ± standard deviation. ADL, activities of daily living; FVC, forced vital capacity; FEV1, forced expiratory volume in 1 s; FEV1/FVC, ratio of FEV1 to FVC; PEF, peak expiratory flow; MVV, maximal voluntary ventilation.

### The results of musculoskeletal ultrasonography measurement

Before treatments, the DT, DTF, and DM were not significantly different between the two groups (Table 2, *P* > 0.05).

**TABLE 2:** DT, DTF, and DM in patients, bilateral chest wall before treatment.

		Control group		Treatment group	
		Affected side	Unaffected side	Affected side	Unaffected side
DT (cm)	CEE	0.20 ± 0.12	0.23 ± 0.17	0.19 ± 0.13	0.23 ± 0.17
	MEI	0.27 ± 0.20	0.34 ± 0.31	0.26 ± 0.19	0.35 ± 0.30
DM (cm)	QB	1.27 ± 0.11	1.46 ± 0.13	1.25 ± 0.10	1.41 ± 0.13
	DB	2.75 ± 0.40	5.08 ± 0.68	2.77 ± 0.41	4.96 ± 0.62
DTF (%)		34.19 ± 6.32	48.99 ± 6.39	34.29 ± 3.89	50.10 ± 7.12

Values are presented as mean ± standard deviation. DT, diaphragmatic thickness; DM, diaphragmatic mobility; DTF, diaphragmatic thickening fraction; CEE, calm end-expiratory; MEI, maximum end-inspiratory; QB, quiet breathing.

**FIGURE 2:**
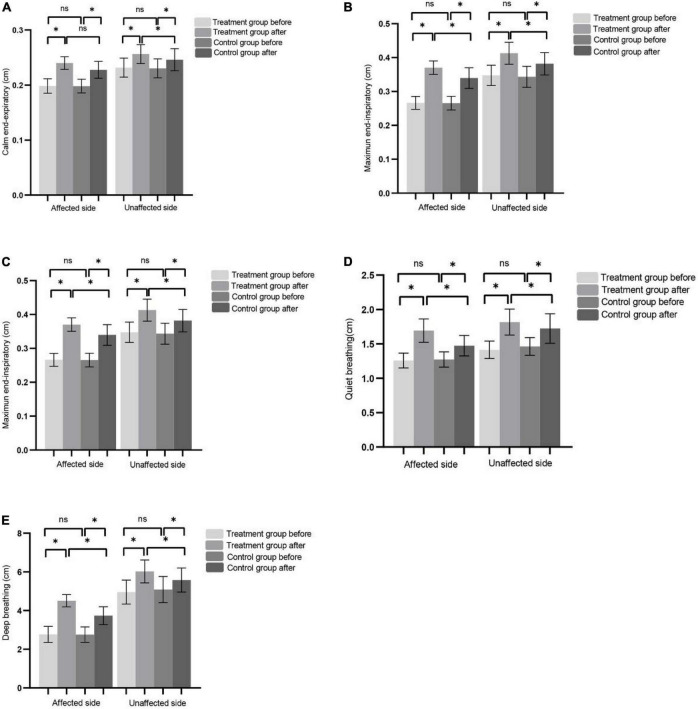
**(A–C)** Represents CEE, MEI, and DTF of the affected side and non-affected side before and after treatment in the control group and the treatment group, respectively. **(D)** The DM of affected side and unaffected side during QB before and after treatment in control and treatment group. **(E)** The DM of affected side and unaffected side during DB before and after treatment in control and treatment group. (*P < 0.05), ns (*P* > 0.05).

After 8 weeks of treatment, the values of DT, DTF, and DM in the affected and unaffected sides in the two groups were significantly improved compared with those before treatment. After treatment, the improvement of DT, DTF, and DM in the affected side and unaffected side in the treatment group was significantly better than that in the control group (Figures 2A–E).

### The results of lung function assessment

**FIGURE 3:**
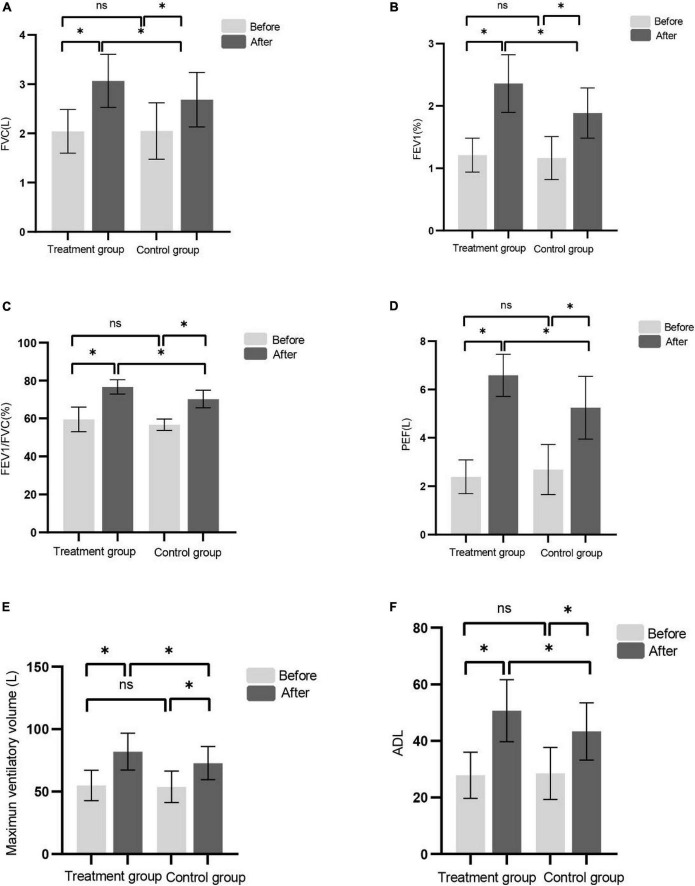
**(A–F)** Shows FVC, FEV1, FEV1/FVC, PEF, MVV, and ADL before and after treatment in the control group and the treatment group, respectively. (*P < 0.05), ns (*P* > 0.05).

We used pulmonary function tester to assess the FVC, FEV1, FEV1/FVC, PEF, and MVV before and after the treatments, respectively. There was no significant difference in the lung function index between the two groups before treatment (*P* > 0.05). Lung function was improved in both groups after treatment. After 8-week treatment, compared with the control group, the pulmonary function test indicators increased more significantly in the treatment group (Figures 3A–E).

### The results of activities of daily living assessments

There was no significant difference in ADL score between the two groups before treatment. After 8 weeks of treatment, the ADL score in the two groups were higher than that before treatment and the effect of the treatment group were better than that of the control group (Figure 3F). Consistent with recent studies (Britto et al., 2011; Ramos et al., 2020).

## Discussion

In our study, both the affected and unaffected sides of DT, DTF, and DM were significantly increased after treatment, and the lung function detection indexes FVC, FEV1, FEV1/FVC, PEF, and MVV all improved significantly. These results indicate that a short time of rTMS combined with RMT training (8 weeks) can improve the lung function in hemiplegic patients with acute ischemic stroke. Cohen found that diaphragm deviation was significantly positively associated with inspiratory volume in hemiplegic patients (Cohen et al., 1994). In our study, the changes in multiple indicators, including DT, DTF, and DM, were consistent with the lung function in hemiplegic patients.

Volitional breathing is controlled by the brain center and located on both sides of the primary motor cortex (Gandevia and Rothwell, 1987). Studies have shown that each hemi-diaphragm is mainly controlled by a unilateral contralateral center. The rTMS also showed that the diaphragmatic response to the stimulation of a single cerebral hemisphere was mainly contralateral, while the ipsilateral response was lower (Maskill et al., 1991). Therefore, in order to better improve the lung function, we stimulated both hemispheres of the brain.

Pulmonary rehabilitation is a central part of the treatment of patients with chronic respiratory diseases (Nici et al., 2012). Stroke patients are often accompanied by respiratory dysfunction, which increases the probability of respiratory tract infection, extends the hospital stay, and brings serious pain to the patients. Therefore, it is vital to promote pulmonary function rehabilitation in stroke. The diaphragm has atrophy after stroke, and it is significantly thinner on the affected side than on the non-affected side at end expiration and TLC (total vital capacity) (Kim et al., 2017; Fabero-Garrido et al., 2022). Which resonates with our findings. Jung et al. (2014) Suggest that diaphragmatic movement is associated with the lung function in stroke patients. Therefore, our aim is to promote the activity of diaphragm, enhance thickness and mobility of diaphragm, promote the recovery of respiratory function, and further promote the functional recovery of stroke patients by using a simple and effective rehabilitation program.

RMT mainly improves the lung function by strengthening peripheral respiratory muscle strength. Transcranial magnetic stimulation can act on the central nervous system and has been widely used. In our paper, we adopted the central plus peripheral mode of respiratory function training to treat stroke patients. The results showed that RMT combined with rTMS could obtain better therapeutic effect.

Ultrasonography can dynamically observe the respiratory muscle function in real time. Ultrasonic probes with different shapes and resolutions can be used to observe the shape and contraction of diaphragm and auxiliary respiratory muscles during calm and deep breathing, including the position, shape, motion amplitude, motion time, acceleration of diaphragm and the thickness changes before and after contraction of diaphragm. Therefore, Ultrasonography was used for evaluation and measurement in this study.

Study has shown that the critical time to begin recovery after stroke is in the first 30 days after stroke (Krakauer et al., 2012), with the majority of patients reached or near to their maximum recovery by 3 months after stroke (Verstraeten et al., 2020). Therefore, we limited the included patients to a stroke course of less than 3 months.

This study also has some limitations. On the one hand, the sample size is not large enough and the research cycle is not long enough. On the other hand, whether rTMS is effective for PD in patients with chronic hemiplegia has not been verified. In the future, we can further study the therapeutic effect of rTMS on patients with chronic hemiplegia. However, this study confirms the therapeutic role of rTMS, which can be used for the rehabilitation of lung function.

## Conclusion

The rTMS combined with RMT can effectively improve the lung function in early hemiplegia patients, which is more effective than RMT alone. Improvement of lung function may be attributed to increased local blood flow during stimulation. Meanwhile, the changes both before and after treatment in DT, DTF, and DM measured by musculoskeletal ultrasonography (MSUS) are consistent with the lung function and can be used as objective indicators in response to lung function. At the same time, the changes of ultrasound index, such as DT, DTF, and DM, as well as lung function index FVC, FEV1, FEV1/FVC, and PEF were consistent with the improvement of patients, activity of daily living. Therefore, we believe that the improvement of pulmonary function can further improve the activity of daily living of stroke patients.

## Data availability statement

The original contributions presented in this study are included in the article/supplementary material, further inquiries can be directed to the corresponding author/s.

## Ethics statement

The studies involving human participants were reviewed and approved by the Department of Physical Medicine and Rehabilitation Dushu Lake Hospital of Soochow University. The patients/participants provided their written informed consent to participate in this study. Written informed consent was obtained from the individual(s) for the publication of any potentially identifiable images or data included in this article.

## Author contributions

All authors listed have made a substantial, direct, and intellectual contribution to the work, and approved it for publication.
